# An economic analysis of a wearable patient sensor for preventing hospital-acquired pressure injuries among the acutely ill patients

**DOI:** 10.1007/s10754-021-09304-7

**Published:** 2021-04-09

**Authors:** Leo Nherera, Barrett Larson, Annemari Cooley, Patrick Reinhard

**Affiliations:** 1 Smith+Nephew, Fort Worth, TX USA; 2 Department of Anesthesia, Smith+Nephew, Texas and Stanford University School of Medicine, Fort Worth, USA; 3 Department of Nursing, San Joaquin Valley College, Bakersfield, CA USA

**Keywords:** Pressure injuries, Hospital-acquired conditions, Prevention, Cost effectiveness, Wearable sensors

## Abstract

More than 2.5 million people in the United States develop pressure injuries annually, which are one of the most common complications occurring in hospitals. Despite being common, hospital-acquired pressure injuries (HAPIs) are largely considered preventable by regular patient turning. Although current methodologies to prompt on-time repositioning have limited efficacy, a wearable patient sensor has been shown to optimize turning practices and improve clinical outcomes. The purpose of this study was to assess the cost-effectiveness of patient-wearable sensor in the prevention of HAPIs in acutely ill patients when compared to standard practice alone. A decision analytic model was developed to simulate the expected costs and outcomes from the payer’s perspective using data from published literature, including a recently published randomized controlled trial. Both univariate and probabilistic sensitivity analysis were conducted. The patient-wearable sensor was found to be cost saving (dominant). It resulted in better clinical outcomes (77% reduction in HAPIs) compared to standard care and an expected cost savings of $6,621 per patient over a one-year period. Applying the model to a cohort of 1,000 patients, an estimated 203 HAPIs would be avoided with annualized cost reduction of $6,222,884 through all patient treatment settings. The probabilistic analysis returned similar results. In conclusion, the patient-wearable sensor was found to be cost-effective in the prevention of HAPIs and cost-saving to payers and hospitals. These results suggest that patient-wearable sensors should be considered as a cost-effective alternative to standard care in the prevention of HAPIs.

## Introduction

In 2019, the Agency for Healthcare Research and Quality reported that HAPIs had increased by 6% over 2014 baseline, whereas all other hospital-acquired conditions (including falls, ventilator-associated pneumonias, and central line-associated bloodstream infections) decreased by an average of 13% over their 2014 baseline rates (Bysshe et al., 2017). According to the AHRQ report, 683,000 HAPIs occurred in 2017 with an excess mortality rate of 0.041 per HAPI. An estimated 28,000 patients died as a direct result of having acquired a pressure injury during their hospitalization. The number of HAPI-related deaths was higher than falls, adverse drug effects, surgical site infections, ventilator-associated pneumonias and central line-associated bloodstream infections combined.

Routine patient repositioning has been shown to strongly correlate with lower incidence of HAPI (Bergquist-Berenger et al., 2013) and is a recommended clinical practice to prevent pressure injuries for all at-risk patients (EPUAP/NPIAP/PPPIA, 2019). Patient repositioning at regular intervals reduces the duration of time tissues are exposed to pressure, hence minimizing the opportunity for a pressure injury to occur. Most recent guidelines recommend repositioning all patients at risk for pressure injuries in a way that offers optimal offloading of all bony prominences and maximizes pressure redistribution (EPUAP/NPIAP/PPPIA, 2019). Traditionally, turn protocols have required adherence to a 2-h repositioning interval, yet multiple studies show that this standard is rarely met. Studies examining adherence to turn protocols have estimated it to be between 10% (Winkelman et al., 2010) and 64% (Schutt et al., 2017).

Traditional prevention efforts and hospital incentives (Padula et al., 2019) have largely failed to rein in pressure injuries, and HAPIs continue to be expensive to treat. Individual HAPI treatment costs range from roughly $5,000 (Spetz et al., 2013)–over $100,000 (Brem et al., 2010), with most recent data suggesting that the mean cost to treat a HAPI is $21,767 per occurrence (Wassel et al., 2020). These costs increase significantly for critical care patients and for more severe pressure injury stages. At the national level, the United States is estimated to spend between $10 billion (Berlowitz et al., 2019) and $26.8 billion (Padula et al., 2019) dollars per year on pressure injury related costs.

## Aims

The aim of this economic evaluation was to determine whether the use of a patient-wearable sensor (LEAF Patient Monitoring System™, Smith + Nephew, Inc.), which has been shown to reduce HAPIs by increasing adherence to turn protocols (Schutt, 2017; Yap, 2019), is a cost-effective prevention strategy when used as an adjunct to standard care, when compared with standard care alone.

## Methods and perspective

A decision-analytic model was constructed to determine the total incremental cost and benefit of the intervention over standard care, compared with standard care alone from a US payor perspective, and if investment in a prevention strategy would save overall costs related to HAPI. The findings are also applicable to long-term care facilities where patients are at risk for pressure injuries and may thus need constant monitoring.

**Fig. 1:**
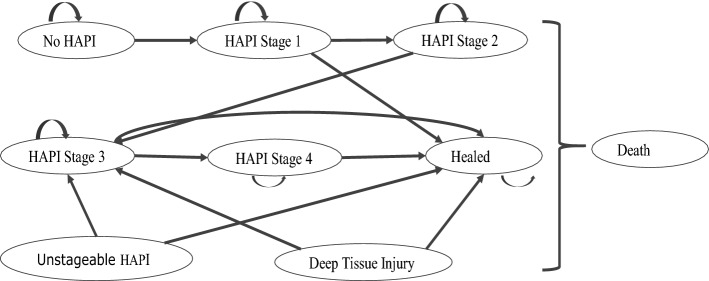
Model flow diagram

Decision models are widely used to determine the cost-effectiveness of one management or treatment strategy compared with an alternative strategy (Hoang et al., 2016; Drummond et al., 2005). We developed a Markov model with nine different health states to estimate the cost-effectiveness and cost utility of a patient-wearable sensor in addition to standard care compared to standard care alone in the prevention of HAPIs. The Markov modelling approach is useful for estimating costs and consequences for recurring chronic conditions that change over time (Hoang et al., 2016; Kuntz et al., 2013). In the model, all patients start in the no HAPI health state when they are admitted in the hospital intensive care unit (ICU) or general ward, and then transition over time to other health states at defined intervals (cycles). This model used time horizons of 52 weeks and a cycle length of 1 week in line with other published economic evaluations (Padula et al., 2011; Pham et al., 2011) in the base case. At the end of week 1, for those that develop HAPIs, a proportion of the cohort moved progressively into either pressure injury stage 1, 2, 3, 4, or had an unstageable HAPI, or developed a deep tissue injury (DTI), healed or died. Figure 1 shows the schematic presentation of the model and the arrows represent transition probabilities from no HAPIs, to stage 4 progressively. Patients can stay in the same health state (stage), as shown by a circular arrow, or move to the next stage. The proportion of patients in each of the different stages of HAPIs was obtained from a study by Pickham et al. (Pickham et al., 2018a). Those that developed HAPIs could also heal or die according to a constant transition probability obtained from the literature (Pickham et al., 2018a; Padula et al., 2011; Padula et al., 2019; Pham et al., 2011). We assumed that patients that developed DTI or unstageable HAPIs, followed a similar path to those patients that developed stage 2 HAPIs. Thus, they would progress to stage 3 HAPI, heal, or die. Death health state is an absorbing state, because patients cannot move out of this state once they enter. Probability of dying was obtained from the US Life Tables (2017), using a 75-year-old with no HAPI in the base case. Wassel et al. (2020) found that patients with HAPIs were at increased risk of mortality by 4.09, 5.09, 5.36, 5.75 and 6.67 times for HAPI stages 1 to 4 and unstageable, respectively. We applied this increased risk of dying to the baseline of 3.05% to calculate the probability of dying for each HAPI stage. Those patients that did not develop HAPIs exited the model upon discharge and are assigned the general cost of no HAPIs.

## Interventions

For the base case analysis, HAPI incidence data were used from the recent 1312-patient randomized controlled trial (RCT), which investigated the effectiveness of using a patient-wearable sensor in addition to standard care compared with standard care alone (Pickham et al., 2018a). The patient-wearable sensor (LEAF^◊^ system, Smith + Nephew, Inc. Fort Worth, TX) provided visual turn cues to care staff based on prescribed repositioning frequency, adequacy of the turn per prescribed turn angle, and reperfusion time between turns. Standard care included adhering to the latest international guidelines on pressure injury prevention (NPUAP/ EPUAP 2019), in addition patients received turning care based on traditional turn reminders and standard practices. Standard care was meant to minimize or eliminate friction and shear, minimize pressure with off-loading, manage moisture, and maintain adequate nutrition and hydration.

The study by Pickham et al. (2018a), found that the use of the patient-wearable sensor was associated with a reduction in HAPI incidence from 2.7 to 0.7%. The study population consisted of patients who were admitted to the ICU with no existing pressure injuries but were deemed to be at risk of developing one. In addition to standard care, patients in the intervention group received optimal turning practices, influenced by real-time data derived from a wearable patient sensor. The patient-wearable sensor reduced the occurance of HAPIs by 73% based on per-protocol analysis or by 67% based on intention-to-treat analysis. Once a HAPI developed, data in the study was not tracked to monitor the impact of the sensor on subsequent transitions between different pressure injury stages. For our sensitivity analysis, data was obtained from a meta-analysis involving 7 hospitals and the Pickham et al. RCT (Nherera et al., 2020), where the HAPI rates of patients receiving the wearable sensor versus standard care were compared. This meta-analysis showed that the patient-wearable sensor reduced the occurrence of HAPIs by 70%. Probabilities of moving from one HAPI stage to another were informed by cost-effectiveness publications from Padula et al. (2019) and Pham et al. (2011).

## Utility data used in the model

The quality of life (QoL) weights in a study by Padula et al. (2011) were used. The weights were measured using EQ-5D and are obtained from the US populations who are in their 50 s. Although health-related quality of life (HRQOL) is dependent on age (Fryback et al., 2007), for simplicity, we did not incorporate this age dependency on the HRQOL estimates used in the model. Table 1 below shows the clinical and utility data used in the model.

**Table 1:** Clinical and utility data used in the model

Description	Mean	Lower value	Upper value	Distribution	References
Incidence (%) of HAPIs with standard of care alone	2.70%	2.30%	3.26%	Beta distribution Alpha = 0.059 Beta = 2.13	Pickham 2018a
*Baseline probabilities*					
Transition probability from HAPI Stage 1 to HAPI Stage 2	1.82%	1.40%	2.82%	Log normal SE = 18.24%	Padula 2019
Transition probability from HAPI Stage 1 to healed HAPI	3.78%	3.22%	4.39%	SE = 8.06%	Padula 2019
Transition probability from HAPI Stage 2 to HAPI Stage 3	1.54%	0.53%	0.70%	SE = 7.49%	Padula 2019
Transition probability from HAPI Stage 2 to healed HAPI	2.94%	2.45%	3.92%	SE = 12.23%	Padula 2019
Transition probability from HAPI Stage 3 to HAPI Stage 4	0.71%	0.19%	1.18%	SE = 47.54%	Pham 2011
Transition probability from HAPI Stage 3 to healed HAPI	0.73%	0.14%	1.11%	SE = 53.9%	Pham 2011
Transition probability from HAPI Stage 4 to healed HAPI	0.23%	0.09%	0.58%	SE = 48.5%	Pham 2011
Transition probability from unstageable HAPI to HAPI stage 4	1.54%	0.53%	0.70%	SE = 7.49%	Assumed to be same as Stage 2
Transition probability from unstageable HAPI to healed HAPI (assumed same transitions as Stage 3 to 4)	2.94%	2.45%	3.92%	SE = 12.23%	Assumed to be same as Stage 2
Transition probability from DTI to HAPI Stage 3	1.54%	0.53%	0.70%	SE = 7.49%	Assumed to be same as Stage 2
Transition probability from DTI to healed HAPI (assumed same transitions as Stage 2 to 3)	2.94%	2.45%	3.92%	SE = 12.23%	Assumed to be same as Stage 2
Relapsed HAPI from healed to HAPI Stage 1 and 2	0.81%	0.69%	0.93%	SE = 7.95%	Padula 2011
Relapsed HAPI from healed to HAPI Stage 3 and 4	0.25%	0.21%	0.28%	SE = 7.58%	Padula 2011
*Mortality*					
HAPI Stage 1 mortality	12.47%	11.35%	13.73%	Beta distribution Alpha = 5.42 Beta = 38.05	Wassel 2020 Life Tables 2017
HAPI Stage 2 mortality	15.52%	14.70%	16.41%	Beta distribution Alpha = 24.72 Beta = 134.49	Wassel 2020 Life Tables 2017
HAPI Stage 3 mortality	16.35%	14.40%	16.41%	Beta distribution Alpha = 4.98 Beta = 25.50	Wassel 2020, Life Tables 2017
HAPI Stage 4 mortality	17.54%	14.82%	20.74%	Beta distribution Alpha = 3.14 Beta = 14.78	Wassel 2020, Life Tables 2017
Unstageable mortality	20.34%	18.27%	22.63%	Beta distribution Alpha = 10.41 Beta = 40.77	Wassel 2020, Life Tables 2017
DTI mortality	20.34%	18.27%	22.63%	Beta distribution Alpha = 10.41 Beta = 40.77	Wassel 2020 Life Tables 2017
No HAPI mortality	3.05%	2.29%	3.81%	Beta distribution, Alpha = 0.021 Beta = 0.652	Wassel 2020 Life Tables 2017
*Intervention effectiveness*					
Hazard ratio of HAPIs Relative risk of PU					
Patients wearable sensor vs standard care	0.27	0.10	0.75	Log normal SE 0.52	Pickham 2018a PP analysis
	0.33	0.12	0.90	SE = 0.52	Pickham 2018a ITT analysis
	0.30	0.21	0.44	SE = 0.19	Nherera 2020
*Utility*					
Utility value of HAPI Stage 1	0.819	0.70	0.94	Beta distribution Alpha = 18.74 Beta = 4.14	Padula 2011
Utility value of HAPI Stage 2	0.778	0.66	0.90	Beta distribution Alpha = 20.81 Beta = 5.94	Padula 2011
Utility value of HAPI Stage 3	0.597	0.51	0.69	Beta distribution Alpha = 22.37 Beta = 15.10	Padula 2011
Utility value of HAPI Stage 4	0.597	0.51	0.69	Beta distribution Alpha = 22.37, Beta = 15.10	Padula 2011
Utility value of unstageable HAPI	0.778	0.66	0.90	Beta distribution, Alpha = 22.37 Beta = 15.10	Assumed same as Stage 2
Utility value of DTI	0.778	0.66	0.90	Beta distribution Alpha = 22.37 Beta = 15.10	Assumed same as Stage 2
Utility value of healed or No HAPI	0.829	0.70	0.95	Beta distribution Alpha = 18.17 Beta = 3.75	Padula 2011
Utility value of death HAPI	0	0.00	0.00	Not varied	Padula 2011

*DTI* deep tissue injury; *HAPI* hospital-acquired pressure injury; *ITT* intention to treat; *PP* per protocol; *SE* standard error of the mean

## Resource costs

The model included the cost of the patient-wearable sensor, which was the 2019 list price obtained from the manufacturer. The cost of standard care was not directly modelled as this was assumed to be the same between the two strategies being compared. The cost of treating admitted patients who developed HAPIs and those who did not develop HAPIs was obtained from matched patients admitted in hospital ICU reported in the study by Wassel et al. (2020), which utilized data from the Premier Healthcare Database (PHD), a US service-level, all-payer database that contains information on medications, laboratory tests performed, diagnostics, and therapeutic services, in statistically de-identified patient daily service records. Wassel et al. (2020), assessed the occurrence of HAPIs using ICD-9 codes 707.00–707.09 and 707.20–707.25., and key outcomes included, readmissions, length of hospital stay (LOS) and costs. The total costs by HAPI stage included all services, medications, and supplies billed during the index hospitalization and factored in the patient's LOS including any days of ICU stay. We adjusted the costs for inflation to 2020 US dollars using the U.S. Department of Labor Consumer Price Index-All Urban Consumers data. The cost of the intervention was calculated using the manufacturer’s 2020 list price for the patient sensor. We also explored the possibility that some patients may need more than one sensor in sensitivity analysis. The costs over the model time horizon defined as 26 weeks and 52 weeks were summed to give a total cost of treatment for each arm of the model. Because of the relatively short timescale, costs and effects were not discounted. The model was implemented using Microsoft Excel (Microsoft Corporation) and the cost inputs are shown in Table 2.

**Table 2:** Cost data applied in the model

ICU costs					
Description	Mean	Lower value	Upper value	Distribution (Gamma distribution)	References
Total cost of episode: Stage 1 HAPI	$54,993	53,503	56,525	Alpha = 5089 Beta = 11	Wassel 2020
Total cost of episode: Stage 2 HAPI	$64,410	63,394	65,442	Alpha = 15,199 Beta = 4	Wassel 2020
Total cost of episode: Stage 3 HAPI	$84,967	82,067	87,970	Alpha = 3184 Beta = 27	Wassel 2020
Total cost of episode: Stage 4 HAPI	$115,894	110,091	122,003	Alpha = 1455 Beta = 80	Wassel 2020
Total cost of episode: Unstageable HAPI	$68,197	65,899	70,575	Alpha = 3269 Beta = 21	Wassel 2020
Total cost of episode: DTI	$68,197	65,899	70,575	Alpha = 3269 Beta = 21	Wassel 2020
Total cost of episode: No HAPI	$36,317	36,251	36,382	Alpha = 1,180,999 Beta = 0	Wassel 2020
*General ward costs*					
Total cost of episode: Stage 1 HAPI	$35,273	34,668	35,888	Alpha = 12,845 Beta = 3	Wassel 2020
Total cost of episode: Stage 2 HAPI	$41,664	41,207	42,126	Alpha = 31,584 Beta = 1	Wassel 2020
Total cost of episode: Stage 3 HAPI	$54,151	52,725	55,617	Alpha = 5388 Beta = 10	Wassel 2020
Total cost of episode: Stage 4 HAPI	$67,198	64,473	70,038	Alpha = 2241 Beta = 30	Wassel 2020
Total cost of episode: Unstageable HAPI	$43,821	42,705	44,967	Alpha = 5767 Beta = 8	Wassel 2020
Total cost of episode: DTI	$43,821	42,705	44,967	Alpha = 5767 Beta = 8	Wassel 2020
Total cost of episode: No HAPI	$20,684	20,662	20,706	Alpha = 3,395,751 Beta = 0	Wassel 2020
*Cost of the intervention*					
Patient-wearable sensor per day	$13.33			Not varied	List price from manufacturer

*ICU* intensive care unit

## Cost-effectiveness analysis

The difference in costs (the incremental cost) was calculated as the total cost for patient-wearable sensor plus standard care minus the total cost for standard care alone. Effectiveness was calculated in two ways: 1) the number of HAPIs avoided at 52 weeks, and 2) the total number of quality adjusted life years (QALYs) gained at 52 weeks. The incremental effectiveness was calculated as effectiveness of patient-wearable sensor plus standard care minus the effectiveness of standard care alone. The incremental cost-effectiveness of the patient-wearable sensor relative to standard care was calculated as the difference between the incremental costs of the patient-wearable sensor divided by the incremental difference in effectiveness between the two strategies measured in HAPIs avoided or QALYS over 52 weeks.

## Sensitivity analysis

Whenever an economic model is done, the model parameters used, such as the various probabilities and unit costs, are subject to uncertainty (O'Brien and Briggs, 2002). In order to investigate the effect of uncertainty in the model assumptions, two types of sensitivity analyses were undertaken. Firstly, a one-way sensitivity analysis, which gives insight into the relative sensitivity of the results to the individual model inputs. This approach varies individual inputs one at a time holding other inputs constant and recording the resultant incremental costs and benefits. Values used in the sensitivity analysis are derived from literature whenever possible; if they are not reported, it is useful to set the limits of each variable to a defined percentage for instance ± 20% of the point estimate, to enable the comparison of sensitivity of inputs.

Recently, one-way sensitivity analysis has become less important because of the widespread development and adoption of probabilistic methods (O'Brien and Briggs, 2002). We therefore implemented a probabilistic sensitivity analysis, where each input is assigned a probability distribution, and the model is run many times, drawing input values from the assigned distributions at random. While the one-way sensitivity analysis changes the input values one at a time, a probabilistic approach changes all the values of the model parameters simultaneously. The distributions used in the probabilistic model and values used in the one-way sensitivity analysis are shown in Tables 1 and 2 and a total of 2,000 iterations of the model were run.

## Scenario analyses

Additional scenario analyses were included as follows: (1) a 26-week time horizon where, instead of the 52 weeks used in the base model; (2) assessed the cost-effectiveness in the general ward instead of the ICU, assuming the occurrence of HAPI was similar in general and ICU units; and (3) time horizon equal to average LOS for a single hospitalization (rounded to 4 weeks).

## Results

The patient-wearable sensor in addition to standard care was found to be cost saving and more effective than standard care alone (Table 3). Switching patient practice from standard care alone to adding a patient-wearable sensor on top of standard care would result in an expected cost saving of $6,621 per patient, and an expected reduction in HAPI incidence of 77% over 52 weeks. Applying the model to a cohort of 1,000 patients, it is estimated that 203 HAPIs would be avoided, with an overall cost reduction of $6,621,113. The patient-wearable sensor, when added to standard care practices for HAPI prevention, is superior to standard care alone from both a clinical and financial perspective.

**Table 3:** Baseline results, expected costs and outcomes for a cohort of 1000 patients at 52 weeks for patient-wearable sensor plus standard care compared with standard care alone

Intervention	Patient-wearable sensor	Standard care	Difference
Costs	$39,579,924	$46,201,037	− $6,621,113
QALYs	15.49	14.54	0.95
HAPI avoided	895	693	203

*HAPI* hospital-acquired pressure injury; *QALYs* quality adjusted life years

## One-way sensitivity and scenario analysis

One-way sensitivity analyses were performed on key model inputs to investigate the impact the independent variation of each input had on: the incremental cost; incremental benefit; and incremental cost-effectiveness ratios (Table 4). The one-way sensitivity analysis showed that the model was not sensitive to all of the assumptions that were used in the model, since none of individual changes in parameters resulted in the reversal of the conclusions. The intervention remained dominant (more effective and cost-saving) throughout the sensitivity analysis, indicating that the findings are quite robust to changes in the values. Even when we explored the possibility of patients needing more than one sensor, in this case applying multiple sensors per patient, the patient-wearable sensor remained cost saving, suggesting the model is not sensitive to this assumption.

**Table 4:** One-way sensitivity analysis results showing savings per 1000 patients

Base case PP RR 0.27 (95% CI 0.10 to 0.75)	-$6,621,113	
One way-sensitivity analysis	Lower value	Upper value
Effectiveness of patient-wearable sensor PP	− $8,506,636	− $1,940,269
Results ITT RR 0.33 (95% CI 0.12 to 0.90)	− **$5,987,365**	
Effectiveness of patient-wearable sensor ITT	− $8,277,489	− $635,260
Results—Meta-analysis RR 0.30 (95% CI 0.21 to 0.44)	− **$6,302,285**	
Effectiveness of patient-wearable sensor meta-analysis	− $7,270,898	− $4,864,581
General ward costs	− $4,636,163	
Follow up 4 weeks	− $974,815	
Follow up 26 weeks	− $4,865,325	
Number of patient sensors = 2	− $6,421,113	

*ICU* intensive care unit; *ITT* intention-to-treat analysis; *PP* per-protocol analysis, *RR* risk ratio

## Probabilistic sensitivity analysis

**Fig. 2:**
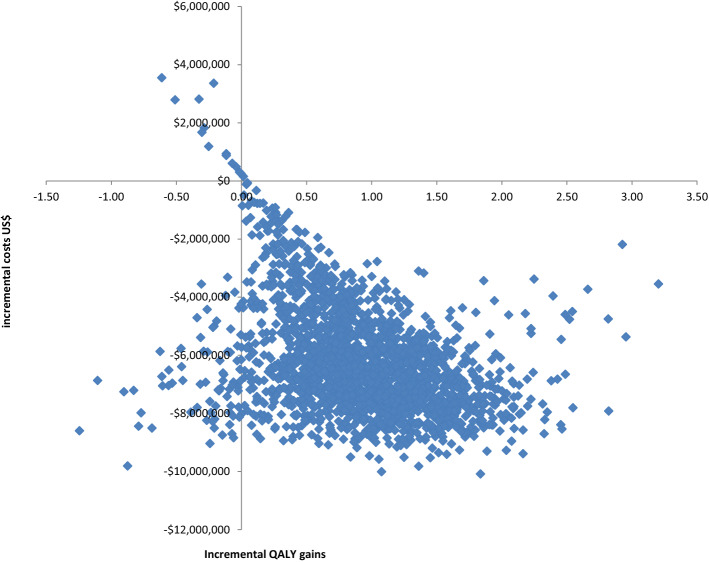
Results of the probabilistic sensitivity analysis; the cost-effectiveness plane

Uncertainty was also assessed using probabilistic analysis. The results of the probabilistic analysis plotted as incremental cost against incremental effectiveness on a plot known as the cost-effectiveness plane are shown in Fig. 2. The patient-wearable sensor is dominant in 96% of the simulations and there is a 99% probability that it is cost-effective if the payer is willing to pay $50,000/QALY gain. Table 5 shows the expected cost per 1,000 patients and number QALYs and HAPIs avoided, obtained from the probabilistic analysis.

**Table 5:** Probabilistic results, expected costs and outcomes for 1000 patients at 52 weeks for patient-wearable sensor plus standard care compared with standard care alone

Intervention	Patient-wearable sensor	Standard care	Difference
Costs	$39,928,973	$46,151,857	− $6,222,884
QALYs	15.49	14.57	0.92
HAPI avoided	885	694	191

*HAPI* hospital-acquired pressure injury; *QALYs* quality adjusted life years

The probabilistic results confirmed the base case deterministic results, suggesting that we can be confident that the patient-wearable sensor is indeed cost saving in preventing HAPIs when compared to standard care alone.

## Discussion

In today’s healthcare systems, it has become increasingly important to demonstrate that new technologies provide financial value. It is therefore important that payers and policymakers have the tools to make the best decisions about which products and services to adopt. One way of making better informed decisions is adopting cost-effectiveness analysis. In the United States, the “Triple Aim” (Institute for Healthcare Improvement) is a framework that aims to optimize the performance of the healthcare system by improving patients’ experience, improving population health, and reducing cost of healthcare per patient (Berwick et al., 2008). This means that for new technologies, their potential to save resources should be considered as well as their clinical effectiveness. Patient-wearable sensors have been shown to be effective in the prevention of HAPIs (Pickham et al., 2018a, b; Nherera et al., 2020) and improves patient repositioning compliance (Yap et al., 2019; Pickham et al., 2018b; Schutt et al., 2017). The study by Pickham et al. (2018a) showed a 73% reduction in HAPIs while the meta-analysis by Nherera et al. (2020) validated the results of the randomised control trial by Pickham et al. (2018a). The meta-analysis by Nherera et al. (2020) consisted of 8 studies which enrolled more than 19,000 patients who used the patient-wearable sensor. The study showed a 70% reduction in HAPIs when compared to standard of care. These studies were conducted in hospitalized acutely ill patients in the United States and therefore the results may not be generalizable to other countries or care settings. Modelling of the results demonstrated that the patient sensor will result in improved clinical benefits in the form of fewer cases of HAPIs (77% reduction in HAPIs) and increased QALYs at reduced costs over a 52-week period. The cost savings are estimated to be $6,621 per patient.

Because the patient sensor results in improved clinical outcomes and is estimated to be less expensive overall, it is deemed to be cost saving or it can be described as a dominant economic strategy. This dominance of the patient-wearable sensor is a result of the increased effectiveness of the sensor in preventing HAPIs. Furthermore, the observed savings could be more than estimated in this study, as it did not take into account other potential costs to the provider, such as litigation costs or intangible costs resulting from poor hospital quality ratings. These results therefore suggest that hospitals and payers would benefit financially, clinically, and reputationally should they choose to implement a patient-wearable sensor.

Considering the budget impact of adopting the sensor for a hospital or payer that has a census of 1,000 patients per year, assuming the effectiveness seen in Pickham et al. (2018a) study and a baseline rate of HAPIs recorded in the same study of 2.7%, we estimate that the patient-wearable sensor will help prevent 20 HAPIs compared to standard care alone (73% reduction, standard care 27 HAPIs vs. 7 HAPIs for patient-wearable sensor in addition to standard of care). Wassel et al. (2020) reported the average additional cost of a HAPI to be $21,767, which varies by HAPI stage. Assuming all the 1,000 patients get the sensor, the hospital can potentially realise a saving of $235,340 per year by adopting this policy. These savings can be more for HAPIs stage 3 and 4 and less for stage 1 and 2.

Our study is the first economic analysis that has considered the use of patient-wearable sonsor in reducing HAPIs. The study is undepinned by robust clinical evidence from a RCT (Pickham et al., 2018a) and further evidence from a meta-analysis (Nherera 2020). HAPIs are a widely recognised problem, the incremental costs of which has been estimated to be about $21,767, varying by stage of HAPI (Wassel et al., 2020). Any intervetion that reduces the occurance of HAPIs will save the payers and hospitals money, as our study has demonstrated. We conducted a number of sensitivity analysis to test the robustness and sensitivity of the model results across a wide range of inputs. We noted that the model was not sensitive to changes in individual inputs, or all of the inputs when varied simultenously. This suggests that we can be confident that the patient-wearable sensor is indeed cost-saving. Prevention of HAPIs is therefore a potentially useful way to reduce healthcare costs and potentially increase profitability of hospitals as well as enhancing reputational status.

In addition to a sensitivity analysis, we also conducted scenario analyses. In the base model we used effectiveness data on the perfomance of the patient-wearable sensor from the per protocol analysis of the RCT by Pickham et al. (2018a). In a scenario analysis, we used the intention-to-treat analysis and also the data from the meta-analysis by Nherera et al. (2020). The results remained robust as we retained the same cost-saving conclusions. The base model was analysed over one year to estimate the annual cost per patient. In sensitivity analysis, we limitted the analysis to 4 weeks in line with the expected length of stay of hospitalised HAPI patients. The model continued to show cost saving of $965 per patient even at 4 weeks. Furthermore the base model considered patients in the ICU, we evaluated perfomance of the patient-wearable sensor in the non-ICU settings, assuming the effect of the sensor was the same in ICU and general care units. Patients in the non-ICU settings accrue less costs compared to those in the ICU as the Wassel et al. study showed. The results remained cost saving, suggesting that we can be confident that the patient sensor is indeed cost saving when compared to standard care in preventing HAPIs.

Our findings are in line with other studies that have considered prevention of pressure injuries before (Padula et al., 2011; Pham et al. 2011). A study by Padula et al. (2011) compared the use of prevention strategy using the Wound, Ostomy, Continence Nurses Society guidelines with standard of care in hospital inpatients and concluded that prevention strategy was cost-saving by about $1,200 per patient. Another study by Pham et al. (2011) evaluated a number of prevention strategies compared to standard of care in long-term care residents where prevention strategies, especially those targeting high-risk patients, were more cost-effective. Our study of course used a different strategy of prevention over and above the best practices that were considered by the other two studies mentioned. This suggests that these current best practices alone are not adequate in preventing HAPIs, as our study and the one by Pickham et al. (2018a) demonstrated.

In addition to the financial benefit and improved clinical patient outcomes, patient-wearable sensors may have additional advantages over standard care not evaluated in the current study. For example, Hendricksen et al. (2019) suggested that the patient-wearable sensors may also reduce hospital-acquired pneumonia by 40% in critical patients (n = 597). Another study by Ifedili et al. (2018) also found that the patient-wearable sensor has the potential to improve teamwork, communication, and compliance with evidence based practice in resident care homes. It is anticipated that this improvement in turning practice, teamwork, compliance and therefore the improvement in clinical outcomes can be sustained as a result of this technology.

## Limitations

This evaluation has several limitations. First, the assumptions made for effectiveness reflect data from only 1 RCT, although more data exists from observational studies that seem to confirm the RCT findings (Nherera et al., 2020). However, we perfomed a sceneraio analysis using data from the meta-analysis and itention-to-treat analysis and we were reassured that the patient-wearable sensor was indeed cost-saving. Second, ICU HAPI occurence data was used in the model assuming that HAPI occurrence is the same in both ICU and general nursing units, despite ICU patients likely being at higher risk for HAPI than patients in general wards due to their prolonged immobility and severity of illness. One occurrence rate was chosen for the model for the sake of simplicity and due to lack of specific occurrence data in general wards. Third, the reported clinical effectiveness did not report by HAPI stage and therefore we assumed the effectiveness of the patient-wearable sensor was the same across all HAPI stages. However, further clinical effectiveness evidence by HAPI stage is needed and the cost-effectiveness model should be updated once such data becomes available. In particular, we recommend that real-world observational studies reporting both clinical and economic data should be conducted as these have the potential to demonstrate the results in non-controlled environments and therefore can be widely generalized to the wider population beyond the acute care setting.

## Conclusions

A decision-analytic model was used to determine the cost-effectiveness of a patient-wearable sensor compared to standard of care in the prevention of HAPIs. The patient-wearable sensor was found to be effective in the prevention of HAPIs and our economic analysis demonstrated that this intervetion is cost-saving to payers and hospitals. These results therefore suggest that the patient-wearable sensor should be considered as a cost-effective alternative to standard care in the prevention of HAPIs. The economic analysis used strong clinical evidence and may provide an opportunity for payers and hospitals to reduce the economic burden of HAPIs. We encourage other scholars to update this model as more clinical evidence becomes available, in particular when the effectiveness data is reported by HAPI stage.

## References

[CR1] Bergquist-Beringer S, Dong L, He J, Dunton N (2013). Pressure ulcers and prevention among acute care hospitals in the United States. Joint Commission Journal on Quality and Patient Safety.

[CR2] Berlowitz D, Lukas CV, Parker V, Niederhauser A, Silver J, Logan C, Ayello E, Zulkowski K. (2019). Agency for healthcare research and quality. Preventing Pressure Ulcers in the Hospitals. A Toolkit for Improving Care. https://www.ahrq.gov/sites/default/files/publications/files/putoolkit.pdf. (Accessed 20 June 2020).

[CR3] Berwick DM, Nolan TW, Whittington J (2008). The triple aim: care, health, and cost. Health Aff (Millwood).

[CR4] Brem H, Maggi J, Nierman D, Rolnitzky L, Bell D, Rennert R, Golinko M, Yan A, Lyder C, Vladeck B (2010). High cost of stage IV pressure ulcers. American journal of surgery.

[CR5] Bysshe, T, Gao, Y., Heaney-Huls, K., Hockenberry, J., Hovey, L., Laffan, A.M., Lee, S., Murphy, D.J., Watts, E.A. (2017). Estimating the Additional Hospital Inpatient Cost and Mortality Associated With Selected Hospital-Acquired Conditions. AHRQ Publication No. 18–0011-EF. https://www.ahrq.gov/hai/pfp/haccost2017.html (Accessed 20 Jun 2020).

[CR6] Drummond M, Sculpher M, Torrance G, O'Brian B, Stoddart G (2005). Methods for the Economic Evaluation of Health Care Programmes.

[CR7] European Pressure Ulcer Advisory Panel, National Pressure Injury Advisory Panel and Pan Pacific Pressure Injury Alliance. Prevention and Treatment of Pressure Ulcers/Injuries:Clinical Practice Guideline. The International Guideline. (2019). Emily Haessler (Ed.). EPUAP/NPIAP/PPPIA.

[CR8] Fryback DG, Dunham NC, Palta M, Hanmer J, Beuchner J, Cherepanov D, Herrington S (2007). US norms for six generic health-related quality-of-life indexes from the National Health Measurement Study. Medical Care.

[CR9] Hendricksen, K; Johnston, S; Jularbal-Walton, J. (2019) Turning Program Reduces Hospital-Acquired Pressure Injuries in Critical Care. Poster presented at the Annual meeting of American Organization of Nurse Executives. 10–13 April 2019; San Diego CA USA

[CR10] Hoang VP, Shanahan M, Shukla N, Perez p; Farrell M; Ritter A.  (2016). A systematic review of modelling approaches in economic evaluations of health interventions for drug and alcohol problems’. BMC Health Services Research.

[CR11] Ifedili IA, Kennerly SM, Sabol VK, Yap TL (2018). Implementing a visual cueing technology intervention in a nursing home: Nursing staff perceptions. Geriatric Nursing.

[CR12] Kuntz, K., Sainfort, F., Butler, M., Taylor, B., Kulasingam, S., Gregory, S., Mann, E., Anderson, JM., Kane, RL. (2013) ‘Decision and simulation modelling in systematic reviews’ rockville (MD): Agency for healthcare research and quality (US); Decision and Simulation Modelling Alongside Systematic Reviews. https://www.ncbi.nlm.nih.gov/books/NBK127482/pdf/Bookshelf_NBK127482.pdf (Accessed 20 April 2020). 23534078

[CR13] Nherera, L., Cooley, A., Delhougne, G.,(2020) Meta-analysis shows patient wearable sensor reduces incidence of hospita- acquired pressure injuries in critically ill patients. Poster presentation at the National Pressure Injury Prevention Panel Annual Conference. 27–28 Febr 2020; Houston, TX, USA.

[CR14] O'Brien BJ, Briggs AH (2002). Analysis of uncertainty in health care cost-effectiveness studies: An introduction to statistical issues and methods. Statistical Methods in Medical Research.

[CR15] Padula WV, Delarmente BA (2019). The national cost of hospital-acquired pressure injuries in the united states. International Wound Journal.

[CR16] Padula WV, Mishra MK, Makic MB, Sullivan PW (2011). Improving the quality of pressure ulcer care with prevention: A cost-effectiveness analysis. Medical Care.

[CR17] Pham B, Stern A, Chen W, Sander B, John-Baptiste A, Thein H, Gomes T, Wodchis WP (2011). Preventing pressure ulcers in long-term care: a cost-effectiveness analysis. Archives of Internal Medicine.

[CR19] Pickham D, Berte N, Pihulic M, Valdez A, Mayer B (2018). Effect of a wearable patient sensor on care delivery for preventing pressure injuries in acutely ill adults: A pragmatic randomized clinical trial (LS-HAPI study). International Journal of Nursing Studies.

[CR18] Pickham D, Pihulic M, Valdez A, Mayer B, Duhon P, Larson B (2018). Pressure injury prevention practices in the intensive care unit: Real-world data captured by a wearable patient sensor. Wounds.

[CR20] Schutt SC, Tarver C, Pezzani M (2017). Pilot study: Assessing the effect of continual position monitoring technology on compliance with patient turning protocols. Nursing open.

[CR21] Spetz J, Brown DS, Aydin C, Donaldson N (2013). The value of reducing hospital-acquired pressure ulcer prevalence: an illustrative analysis. Journal of Nursing Administration.

[CR22] United States Period Life Table 2017. https://www.ssa.gov/oact/STATS/table4c6.html (Accessed 22 September 2020).

[CR23] Wassel CL, Delhougne G, Gayle JA, Dreyfus J, Larson B (2020). Risk of readmissions, mortality, and hospital-acquiredconditions across hospital-acquired pressure injury (HAPI) stages in a US National Hospital Discharge database. International Wound Journal.

[CR24] Winkelman C, Ling-Chun C (2010). Manual Turning in Patients Receiving Mechanical Ventilation. Critical Care Nurse.

[CR25] Yap TL, Kennerly SM, Ly K (2019). Pressure injury prevention: outcomes and challenges to use of resident monitoring technology in a nursing home. Journal of Wound, Ostomy, and Continence Nursing.

